# Supplementary material for: Qualitative study exploring the design of a patient-reported symptom-based risk stratification system for suspected head and neck cancer referrals: protocol for work packages 1 and 2 within the EVEREST-HN programme
Source: BMJ Open. 2024 Apr 5;14(4):e081151. doi: 10.1136/bmjopen-2023-081151 (PMC11002383; doi:10.1136/bmjopen-2023-081151)
Supplement: Supplementary data [file bmjopen-2023-081151supp001.pdf]

Appendix 1. Patient interview topic guide

EVEREST-HN

Using patient-reported symptoms to guide referral for suspected head and neck cancer

PATIENT INTERVIEW TOPIC GUIDE

Version 1.0

EVEREST-HN: Patient Interview Topic Guide

Note: The interview schedule is developmental. The questions will need to be tailored to the specific answers of each interviewee. The interview schedule given here is therefore a general topic guide for the one-to-one qualitative interviews.

Welcome and Introduction.

Ask if any questions. Obtain informed consent

Re-cap of Research and Plan for Interview

Brief re-cap on the aims and purpose of the interview and explain what will happen.

The following questions need not be covered in this particular order but rather the interview should flow as freely and naturally as possible. The interviewer will prompt as appropriate with phrases such as 'can you tell me a little more about that', 'can you give me an example of that', 'how did/do you feel about that'.

Experience of cancer pathway

It may be upsetting for the patient to talk about some parts of their experience. It is important to give them time to talk about this. Modify the questions about their pathway on the basis of what they say about their overall experience.

- **Can you tell me a bit about the symptoms that you have had in your head or neck and how you came to be referred for an urgent hospital appointment?**
    - Symptoms – how long, severity etc
    - Explore what they mean by terms “can you tell me a bit more about that?” “what do you mean by xxx?”
    - What prompted GP consultation (in some people it could have been picked up opportunistically at a routine dental appointment)
    - What did the GP say to you about why they were referring you to hospital?
    - How did you organise the time to go to the hospital?
        - Choice?
- **How did you feel about being referred for an urgent hospital appointment?**
    - Pleased, anxious?
- **How did you find the period while you were waiting for the hospital appointment?**
    - Anxious?
    - Information searching?
- **Can you tell me what happened at your hospital appointment?**
    - Who was there?
    - Tests etc
- **What has happened since that first hospital appointment?**
    - Tests

The EVEREST study is aiming to improve the urgent cancer referral pathway. It would be good to know from your point of view what was good about your experience and what could be improved.

- **What things were good about your experience of an urgent referral?**

- **What things could have been better?**

EVEREST

The next questions are about one idea to improve the urgent cancer referral pathway for patients. We would like to hear what you think of it, as someone who has been through an urgent cancer referral recently.

The idea is that patients would be asked some questions about their symptoms either on a computer or on the phone. This would be in the time between seeing

the GP (or dentist) and going to the hospital. The questions might be about symptoms like a sore throat, a blocked nose or a lump in your neck. The answers would be given to the hospital doctors to look at before they see the patients. Depending on the answers they give, some people might be sent for tests straight away rather than waiting to see a doctor first.

Thinking about people like you who have an urgent referral, how do you think people would feel if they were asked to answer some questions about their symptoms on a computer or on the phone?

- Do you use a computer/smart phone?
- Do you ever use a computer or phone for things like shopping, booking tickets or checking health information (e.g. NHS 111)?
- How do you feel about using the computer for things like that?
  - Convenient or hard work/stressful?
  - Are there any websites that you can think of that are particularly bad or good?
- How do you feel about the idea of answering questions from the hospital about your health on a computer or phone?
- Do you think it would have made you feel more or less anxious to be asked to answer some questions?
- Is there anything that you think might help patients to answer the questions?
- How do you feel about the idea that a computer might help decide whether you were sent straight for tests before you saw a doctor?
  - Do you think it is ok for a computer to help with those decisions or do you think it should always be the doctor?
    - What if the computer made it quicker to get an answer about whether you have cancer or not?

I'd like to ask you about being invited to take part in the EVEREST-HN study. Can you tell me how you were first informed about the study?

- Who was there?
- What else was going on in the appointment?
- Can you remember what they said?
- Can you remember what your first reaction was?

How did you decide whether to take part in the study?

- How did you make your decision?
- What things were important to you?
- Did you speak to other people about the decision? If so, who?
- When did you make the decision?
  - Immediately/nurse phone call/recruitment discussion
- Did you find it an easy decision to make? Why (not)?

Do you remember being given some written information about the study?

- What did you think about that information?
- Was it helpful?
- How did you use it?
- Do you have any suggestions for how it could be improved? If so, how?

Would you have liked information about anything else?

- If so, what?
- Why was that important to you?

Anything Not Covered?

Is there anything that we haven't covered in the interview that you think we should know or think about?

Closing and Thanks

Conclude the discussion and thank the participant for their time and contribution.
