# Supplementary material for: Qualitative study exploring the design of a patient-reported symptom-based risk stratification system for suspected head and neck cancer referrals: protocol for work packages 1 and 2 within the EVEREST-HN programme
Source: BMJ Open. 2024 Apr 5;14(4):e081151. doi: 10.1136/bmjopen-2023-081151 (PMC11002383; doi:10.1136/bmjopen-2023-081151)
Supplement: Supplementary data [file bmjopen-2023-081151supp002.pdf]

Appendix 2. Clinician interview topic guide

EVEREST-HN

Using patient-reported symptoms to guide referral for suspected head and neck cancer

STAFF INTERVIEW TOPIC GUIDE

Version 1.0

EVEREST-HN: Staff Interview Topic Guide

Note: The interview schedule is developmental. The questions will need to be tailored to the specific answers of each interviewee. The interview schedule given here is therefore a general topic guide for the one-to-one qualitative interviews.

Welcome and Introduction.

Ask if any questions. Obtain informed consent

Re-cap of Research and Plan for Interview

Brief re-cap on the aims and purpose of the interview and explain what will happen.

The following questions need not be covered in this particular order but rather the interview should flow as freely and naturally as possible. The interviewer will prompt as appropriate with phrases such as 'can you tell me a little more about that', 'can you give me an example of that', 'how did/do you feel about that'.

Cancer diagnostic pathway

Initial questions about the referral pathway at the specific trust may not be needed if they have been covered in previous interviews.

Can you tell me about how the head and neck urgent cancer referral pathway is organised at the Trust?

- ***Establish in detail (who (including involvement of Nurses and AHPs), how, when):***
  - ***how referrals are processed***
  - ***how clinics are organised***
  - ***how onward referrals are made***

I'd like to know about changes that happened in the referral pathway during COVID-19 – how did you manage urgent referrals during that period?

- ***Did they use any means of triaging referrals? – if so find out about this in detail and about their experiences of this.***

Can you tell me about your role within the head and neck urgent referral cancer pathway?

The EVEREST study is aiming to improve the diagnostic pathway for urgent head and neck cancer referrals. It would be good to know from your point of view what is good about the current pathway and what could be improved.

- **What things do you think are good about the current diagnostic pathway for urgent referral?**
- **What things could be better?**
- **Are there particular sub-groups of patients that are more straightforward or harder to assess?**

You probably remember that we observed/recorded your consultation with (name). Would it be ok if we talked a bit about the consultation so that we can understand more about the processes that were happening?

Specific questions about e.g. why they pursued a particular line of questioning, or what triggered a decision to request a particular test

The EVEREST study is planning to introduce an intervention within the diagnostic pathway. Patients will be asked some questions about their symptoms either on a computer or on the phone. This would be triggered by the receipt of the referral at the hospital. Their answers would be used to calculate a risk score which would be made available to the hospital team, along with any additional information provided by the patient. We hope that this might enable the hospital team to make some decisions ahead of a hospital appointment, e.g. about tests or who needs to be seen most quickly and to provide relevant information in a standardised and accessible format.

What do you think of this idea?

- ***Feasible? (digital exclusion)***
- ***Useful? (an improvement? Would they trust a risk score?)***
- ***How would you see it working? (who might be involved – what roles might change? Who would need to be involved in planning for change?)***
- ***Add to or reduce workloads?***
- ***Is this the best way to improve the pathway or is there something that you think would work better?***

Anything Not Covered?

Is there anything that we haven't covered in the interview that you think we should know or think about?

Closing and Thanks

Conclude the discussion and thank the participant for their time and contribution.
