# Supplementary material for: Qualitative study exploring the design of a patient-reported symptom-based risk stratification system for suspected head and neck cancer referrals: protocol for work packages 1 and 2 within the EVEREST-HN programme
Source: BMJ Open. 2024 Apr 5;14(4):e081151. doi: 10.1136/bmjopen-2023-081151 (PMC11002383; doi:10.1136/bmjopen-2023-081151)
Supplement: Author's manuscript [file bmjopen-2023-081151.draft_revisions.pdf]

BMJ Open is committed to open peer review. As part of this commitment we make the peer review history of every article we publish publicly available.

When an article is published we post the peer reviewers' comments and the authors' responses online. We also post the versions of the paper that were used during peer review. These are the versions that the peer review comments apply to.

The versions of the paper that follow are the versions that were submitted during the peer review process. They are not the versions of record or the final published versions. They should not be cited or distributed as the published version of this manuscript.

BMJ Open is an open access journal and the full, final, typeset and author-corrected version of record of the manuscript is available on our site with no access controls, subscription charges or pay-per-view fees (http://bmjopen.bmj.com).

If you have any questions on BMJ Open's open peer review process please email info.bmjopen@bmj.com

BMJ Open

A qualitative study exploring the design of a patient-reported symptom-based risk stratification system for suspected Head and Neck cancer referrals: protocol paper of work packages 1 and 2 within the EVEREST-HN programme

Journal:	*BMJ Open*
Manuscript ID	bmjopen-2023-081151
Article Type:	Protocol
Date Submitted by the Author:	19-Oct-2023
Complete List of Authors:	Albutt, Abigail; University of Leeds, Leeds Institute of Clinical Trials Research<br>Hardman, John; Northwick Park Hospital<br>McVey, Lynn; University of Bradford, Centre for Digital Innovations in Health & Social Care; Wolfson Centre for Applied Health Research<br>Odo, Chinasa; University of Bradford, Centre for Digital Innovations in Health & Social Care; Wolfson Centre for Applied Health Research<br>Paleri, Vinidh ; The Royal Marsden NHS Foundation Trust<br>Patterson, Jo; University of Liverpool<br>Webb, Sarah; The Royal Marsden NHS Foundation Trust<br>Rousseau, Nikki; University of Leeds, Leeds Institute of Clinical Trials Research<br>Kellar, Ian; The University of Sheffield, Department of Psychology<br>Randell, Rebecca; University of Bradford, Centre for Digital Innovations in Health & Social Care; Wolfson Centre for Applied Health Research
Keywords:	Head & neck tumours < ONCOLOGY, Health informatics < BIOTECHNOLOGY & BIOINFORMATICS, Clinical Decision-Making, QUALITATIVE RESEARCH, Risk Factors, Surveys and Questionnaires

SCHOLARONE™
Manuscripts

A qualitative study exploring the design of a patient-reported symptom-based risk stratification system for suspected Head and Neck cancer referrals: protocol paper of work packages 1 and 2 within the EVEREST-HN programme

Authors and Affiliations

1. Abigail Albutt, Clinical Trials Research Unit, Leeds Institute of Clinical Trials Research, University of Leeds, Leeds LS2 9JT A.K.Albutt@leeds.ac.uk
2. John Hardman, Northwick Park Hospital, Watford Road, Harrow HA1 3UJ johncharles.hardman@nhs.net
3. Lynn McVey, Centre for Digital Innovations in Health and Social Care, Faculty of Health Studies, University of Bradford, Bradford BD7 1DP l.mcvey@bradford.ac.uk
4. Chinasa Odo, Centre for Digital Innovations in Health and Social Care, Faculty of Health Studies, University of Bradford, Bradford BD7 1DP c.r.odo@bradford.ac.uk
5. Vinidh Paleri, The Royal Marsden NHS Foundation Trust, Fulham Road, London SW3 6JJ Vinidh.Paleri@rmh.nhs.uk
6. Joanne M Patterson, School of Health Sciences, Institute of Population Health, University of Liverpool, Liverpool L69 3GB joanne.patterson@liverpool.ac.uk
7. Sarah Webb, The Royal Marsden NHS Foundation Trust, Fulham Road, London SW3 6JJ sarah.webb2@rmh.nhs.uk
8. Nikki Rousseau, Clinical Trials Research Unit, Leeds Institute of Clinical Trials Research, University of Leeds, Leeds LS2 9JT N.Rousseau1@leeds.ac.uk
9. Ian Kellar, Department of Psychology, The University of Sheffield, Sheffield, S1 2LT i.kellar@sheffield.ac.uk
10. Rebecca Randell*, Centre for Digital Innovations in Health and Social Care, Faculty of Health Studies, University of Bradford, Bradford BD7 1DP r.randell@bradford.ac.uk

*Corresponding author

Authors' contributions

JH, IK, JP, VP, NR, RR, Chris Elkington (CE) and John Holmes (JH) (experts by experience) conceptualised the study. NR leads WP1, RR and IK lead WP2; IK, RR and NR are joint senior authors of this manuscript. VP and JP are joint chief investigators for the EVEREST programme; JH is deputy chief investigator for the EVEREST programme. All authors and CE and JH contributed to protocol development. AA wrote the first draft of the manuscript, IK, LMcV, CO, JP, VP, NR, RR, SW reviewed and contributed to subsequent drafts. All authors reviewed and approved the final draft of the manuscript. NR, IK and RR are joint final authors.

Funding statement

This work was supported by NIHR Programme Grants for Applied Research (grant number: NIHR202862).

Competing interests

The authors declare that they have no conflict of interest.

For peer review only

Word count: 3,486.

Abstract

Introduction

Patients presenting to their general practitioners with symptoms of suspected Head and Neck Cancer (sHNC) may be referred to secondary care for a consultation with a specialist. In England, there was an increase in patients referred with sHNC from 140 to 404 patients per 100,000 population between 2009/10 and 2019/20. One in ten patients are not seen within the two-week target and the waiting period leads to significant patient anxiety. Between 2018 and 2020, only 61% of patients diagnosed with head and neck cancer commenced treatment within 62 days of referral. We will develop and implement a pathway for sHNC referrals, based on the Head and Neck Cancer Risk Calculator, to capture patient-reported symptoms. The EVEREST-HN Programme comprises six interlinked work packages. This protocol paper describes the first and second work packages.

Aims and objectives

Work package 1 (WP1) aims to obtain an understanding of language to optimise a patient-reported symptom inventory for sHNC referrals, and outline requirements for the SYmptom iNput Clinical (SYNC) system. Work package 2 (WP2) aims to co-design key elements of the SYNC system, including SYNC symptom questionnaire, and behaviour change intervention materials to integrate the SYNC system into existing hospital workflows.

Methods and analysis

WP1 will use qualitative methods including interviews, observation and recordings of consultations. Rapid qualitative analysis and inductive thematic analysis will be used. WP2 will use focus groups, think aloud technique and experience-based co-design, and will be analysed using qualitative and quantitative approaches.

Ethics and dissemination

This protocol has been reviewed by the Committee for Clinical Research at the Royal Marsden, a Research Ethics Committee and the Health Research Authority. The ethical issues of working with patients on an urgent cancer diagnostic pathway have been considered. Findings will be disseminated via journal publications, conference presentations and public engagement activities.

Key words: diagnosis; head and neck cancer; pathway; referral; risk calculator.

Strengths and limitations

1. This study forms part of a large multicentre study (the EVEREST-HN programme) that will provide evidence about whether the implementation of a patient-reported symptom-based risk stratification system for sHNC referrals is safe, improves patient experience, leads to faster diagnosis and optimises healthcare resource use.
2. A scoping review of the literature and data from two PhD theses were used to generate an initial list of Head and Neck cancer (HNC) symptoms.
3. The study has substantial patient, public and other stakeholder involvement throughout all phases including patients with lived experience of HNC, members of the public without prior

For peer review only

experience of the urgent HNC pathway and hospital staff involved in the HNC diagnostic pathway.
4. A key strength of the study is the use of multiple methods (observation, interviews, think-aloud techniques, experience-based co-design) and theory (Theoretical Framework of Acceptability; Theoretical Domains Framework and Normalisation Process Theory) to inform the development of patient-reported symptom-based risk stratification system.
5. Our approach enables an in-depth exploration from multiple perspectives – but this means sacrificing breadth - only a small number of geographical locations are included.

Introduction

In healthcare, the 'head and neck' region includes the nose, mouth, throat, voice box, thyroid and salivary glands. Many patients present to GPs with symptoms affecting these areas, including hoarse voice, throat discomfort, neck lumps, mouth ulcers and difficulty swallowing. In some patients, these symptoms may be caused by a 'head and neck cancer' (HNC), so, they will be referred to the hospital for an urgent specialist opinion. In England in 2020, 228,482 patients were referred with sHNC, making it the fifth largest group of suspected cancer referrals. After specialist assessment, the vast majority (95%) of these patients can be reassured of being cancer-free, but about 5% will be diagnosed with cancer. Standard practice in the UK is for all sHNC referrals to be offered a face-to-face consultation as their first hospital contact. Unfortunately, partly due to capacity issues, one-in-ten sHNC referrals are not seen within the two-week target.[1] Patients have reported significant anxiety in this period making any delays very undesirable.[2]

The Head and Neck Cancer Risk Calculator (HaNC-RC) symptom inventory was developed for use by specialists. The tool was developed for risk assessment to aid referral of high-risk patients to urgent specialist clinics as patients are currently seen in a chronological order.[3] HaNC-RC-v2 was deployed as a national service evaluation of sHNC referrals undergoing remote triage in secondary care during the initial peak of the coronavirus disease 2019 pandemic.[4] Clinicians were instructed to consider both the clinical history and the outcome from HaNC-RC-v2 to decide on the management plan. Clinician-led telephone triage of 4,568 referrals was recorded:[10] 53.2% were assessed urgently; 16.4% were discharged directly and 68.7% were classed as low-risk using HaNC-RC-v2. Cancer was reported in 5.6% (n=254/4,557) and late cancers were reported in 0.4% despite urgent assessment. This work demonstrated clinicians could perform symptom-based remote triage, supported by risk stratification, medium term outcomes were acceptable and even patients undergoing urgent assessment could have missed cancers.[4] The rapidity of progression of HNC is demonstrated by the impact delays to treatment have on outcomes. It is estimated a one-month delay to treatment led to 437, 514, and 1,415 life years lost for oropharynx, larynx and oral cavity cancers respectively.[5] With increasing numbers of referrals, an improved pathway that identifies people at higher risk for rapid assessment could offer significant benefits.[6]

The EVEREST-HN programme aims to develop and evaluate a patient-reported symptom-based risk stratification system as part of a new pathway for sHNC referrals. Patients (with help from family/carers where needed), will be asked to complete an electronic questionnaire about their symptoms soon after referral. Building on our previous work, we will develop a risk stratification system for HNC based on thousands of new referrals. Hospital staff will review the information from the symptom questionnaire and the patient's individualised risk score to advise the most appropriate management before the patient comes to hospital. Obtaining symptom information from patients before they attend hospital, rather than waiting until their urgent specialist appointment, allows for earlier risk stratification. Using this system, higher-risk patients may have targeted investigations arranged directly, before being seen in clinic, and lower-risk patients may avoid unnecessary investigations before being reassured.

For peer review only - http://bmjopen.bmj.com/site/about/guidelines.xhtml

Patients vary in their confidence in describing their symptoms[7] and so, when taking the patient history, clinicians use accessible and easily interpreted questions. Therefore, it is important the questions used to elicit patient-reported symptoms are understandable to patients. Development of the EVEREST-HN pathway must be based on a comprehensive understanding of existing HNC diagnostic pathways and what patients and clinicians value. Work package 1 (WP1) aims to understand: how clinicians ask questions and decide subsequent steps for patients referred with sHNC; the language patients and clinicians use to describe symptoms;[8] how clinicians reassure and discharge low-risk patients; patients' and clinicians' views of the current diagnostic process for HNC. Work package 2 (WP2) aims to co-design key elements of the SYmptom iNput Clinical (SYNC) system, including the SYNC symptom questionnaire and behaviour change intervention materials to integrate the system into existing hospital workflows. Table 1 describes the subsequent work packages (3-6) of the EVEREST-HN programme and demonstrates how WP1 and WP2 integrate into the wider programme of work.

Table 1. Brief description of EVEREST-HN programme work packages (WP) 3-6.

**Work package**	**Brief description**
3: Informatics linkage of national datasets for suspected HNC referrals	To use data from routinely collected electronic health records to describe the sHNC population and their diagnostic pathway, and provide prognostic information for WP4 and 6.
4: SYNC system development phase-3: Feasibility study to finalise EVEREST-HN pathway	To conduct a concurrent data collection exercise and feasibility study, using the SYNC symptom questionnaire developed in WP1 and 2, alongside the prognostic data from WP3, to develop the SYNC risk stratification algorithm, agree management thresholds and recommendations by stakeholder consensus, finalise all elements of the EVEREST-HN pathway.
5: Cluster randomised controlled trial	To conduct a cluster randomised-trial, with 6-month pilot, to compare the new pathway to standard care, using a primary outcome of cancer diagnosed within 62 days.
6: Health economics	To evaluate the cost-effectiveness of the pathway.

Methods and analysis

WP1- Qualitative research to generate SYNC symptom questionnaire

Study design

WP1 will employ qualitative methods including interviews, observation and recordings of consultations, and analysis of referral documentation. Work will be conducted at three sites with variation in service delivery models, including those not traditionally involved in research. Sites will be recruited to ensure a broad mixture of social, economic and cultural backgrounds of potential participants. Approximately 150 adults (n=50 per site) referred for sHNC, and 15 clinicians (n= 5 per site including different subspecialties that see sHNC patients), will be recruited. A subset of recruited patients (approx n=30) will be interviewed. Clinicians (n=15 varying grades and specialty) will be

For peer review only

interviewed and interviews may include a review of extracts of recordings of their consultations to understand actions taken within and as a result of the consultation. Data collection started in April 2023 and is due to be completed in April 2024.

Sample

Sites will be chosen for variation in service delivery model and to facilitate diversity in patient characteristics. Patient recruitment will aim for variation in age, ethnicity, socio-economic status, presenting symptoms. Clinicians recruited will be clinical staff involved in HNC diagnosis, varying in seniority (consultant, registrar) and specialty (ear, nose and throat surgery, oral and maxillofacial surgery).

Inclusion Criteria
Patients: adults ≥ 18 years referred via sHNC pathway without previous history of HNC. Participant is willing and able to give informed consent for participation in the study.

Clinicians: Staff at participating sites involved in the diagnostic pathway for people with sHNC.

Exclusion Criteria
Individuals who do not meet the inclusion criteria.

Methodology

Consent
Members of the local clinical team will proactively identify eligible patients through triage of referral letters. Eligible patients will be those allocated to attend a diagnostic clinic, or with symptom(s) or other characteristics identified as being important to include in the sample. Where possible, the Patient Information Sheet will be sent to patients with appointment details.

Participants will be approached and consented by local care teams, who will also confirm eligibility. Written consent for consultations to be recorded will be taken immediately prior to appointments. Where patients are willing, consent will also be taken for their contact details to be shared with the research team, so they can potentially be invited to participate in an interview. Verbal consent will be taken and recorded prior to interviews conducted remotely.

The EVEREST-HN qualitative researchers will conduct a small number of clinic observations and qualitative research with participants who have consented to be involved. For clinic observations, informed consent will be received for both clinician and patient participants.

Data Collection

Data collection will comprise:
- Non-participant observation and audio-recordings of diagnostic consultations.
- Review of referral documentation
- Patient interviews
  - the focus will be on their experience of the current diagnostic pathway - we will also seek their views on the EVEREST-HN pathway.
- Clinician interviews.
  - the focus will be on the clinical decision-making process, including views on the EVEREST-HN pathway.

For peer review only

Our recruitment strategy has been co-developed with Patient and Public Involvement (PPI) and with reference to NIHR INCLUDE guidance[9]. Recruitment information will be available in different formats and languages. Topic guides for the interviews will be informed by our own and other research, our PPI and by our theoretical framework including Theoretical Framework of Acceptability (TFA)[10] and Normalistion Process Theory (NPT)[11]which can help to sensitise researchers to relevant aspects of context.[12] (See Appendix 1 and 2 for patient and clinician interview topic guides).

Dataset and case report form (CRF)
Limited descriptive information will be captured to monitor included participants' characteristics, consider sub-groups in the qualitative analysis, and describe participants in study outputs.

The following fields will be recorded on the CRF:

- Demographics (age, gender, ethnicity)
- Referral criteria (e.g., throat lump - based on standard referral forms)
- Smoking status
- Outcome of initial consultation (discharge, further tests, referral to other service)
- Diagnostic outcome (HNC diagnosed, other/no cancer diagnosis)

Pseudonymisation
Sites will generate a unique study ID for each participant and use a 'key' to reference this to the NHS and hospital medical record number. This key will be stored locally at contributing trusts on an excel file on the hard drive of a secure NHS computer. The study key will be stored for the duration of the study and then destroyed in line with local processes for handling patient identifiable data.
Study ID will comprise a single letter hospital code followed by a three-digit consecutive number, e.g. A001, A002, etc.

Theoretical framework
Data collection and analysis will be informed by our theoretical framework which includes TFA and NPT.[10,11] TFA consists of seven constructs: affective attitude; burden; ethicality; intervention coherence; opportunity costs; perceived effectiveness; and self-efficacy.[10] It can be used to highlight characteristics of healthcare interventions that could be improved. NPT considers factors that affect implementation in four areas: coherence; cognitive participation; collective action; reflexive monitoring.[11] NPT has been widely used in studies on implementation of interventions in health care including in trial process evaluations,[13,14] e-health[15] and decision aids.[16,17] In e-health its use has highlighted the importance of sense-making (users' understanding of the e-health intervention's purpose and its differences from usual practice), the impact of digital interventions on roles and responsibilities and the work that must be done to incorporate e-health interventions into pathways.[15,18] Our theoretical framework will be reviewed regularly throughout WP1 and WP2. We will consider whether the existing framework fits the data collected, and whether additional theories, such as the non-adoption, abandonment, scale-up, spread, and sustainability (NASS) framework[19] would be helpful.

Data Storage

Study Database
Descriptive information about patients and their health will be entered by site staff into MACRO, a secure database, for transfer to and storage at the University of Leeds. Participants will be identified by their unique study ID.

For peer review only

Qualitative data will be transcribed and anonymised. The original recordings will be stored securely separately from transcripts and retained for a minimum of 5 years after project-end for data verification, if required. Contact information will be stored until it is no longer needed to contact participants.

Anonymised transcripts and associated pseudonymised descriptive information about participants will be retained after the end of the study to enable data sharing. It will be possible to link the transcript to the descriptive information via the study ID, as this is likely to be essential information as part of data sharing, but after the contact information has been deleted it will not be possible for anyone to link the anonymised information to any identifiable information.

Data Analysis
All interviews will be audio-recorded and transcribed verbatim. Both transcripts and contemporaneous field notes from non-participant observation in clinical settings will be edited to ensure participant anonymity. Analysis will proceed concurrently with data collection using a rapid qualitative analysis approach and will be conducted according to standard procedures of rigorous rapid qualitative analysis [20,21]. Separate Rapid Assessment Procedure (RAP) sheets[20] will be developed for patient interviews, clinician interviews, and consultations based on the research questions. Researchers will independently complete RAP sheets after each interview and for each recorded consultation. We will hold 'data clinics' where the research team exchanges interpretations of key issues emerging from the data. These will facilitate decisions regarding data saturation.[22]

Content analysis of consultation recordings will focus on language used, generating a glossary of terms used by patients to describe their symptoms, and aiming to identify language used by clinicians which is helpful in generating relevant responses from patients, as well as terms that seem to be poorly understood. Thematic analysis of interviews will inform guiding principles (intervention objectives and key design features) and be used to develop a requirement specification and "personas" for co-design. The requirement specification will detail both functional requirements (what the SYNC system should do) and non-functional requirements (including look and feel, usability, performance, and maintainability and support requirements, and requirements relating to implementation).[23] The requirements will be presented using the Volere format.[24]

<u>WP2- Co-design of the SYNC symptom questionnaire</u>

Study design
The co-design process will be iterative, and will involve a series of interactive focus groups, think aloud technique[25] and experience-based co-design (EBCD).[26]

Sample
An ethnic diverse group including participants with English as a second language, will be recruited from PPI groups at the University of Bradford and their networks. We will also involve the EVEREST-HN PPI representatives, to provide their experience of the HNC pathway. Based on previous experience of co-design, we aim to have eight participants.

Methodology

Data collection and analysis

Focus groups
These will be undertaken via Microsoft Teams and recorded. Co-design requires careful preparation but also flexibility, to allow participant involvement in the design of the approach and the

For peer review only

intervention.[27] While open to revision and further specification, we will move from discussing personas and gathering participants' perspectives on what the questionnaire should look like, to discussion of a paper-based prototype to encourage further design input, and discussion of a high fidelity prototype, in terms of how comprehensible, usable, and acceptable it is. Throughout the focus groups the moderator will encourage discussion of differences of opinions.[28] At the end of each focus group, the moderator will present any tentatively identified issues to participants for confirmation or clarification.[29]

Audio recordings will be transcribed verbatim. Data analysis will be conducted after each focus group using thematic analysis and will contribute towards refinements to the requirement specification and determine priorities for subsequent prototypes. At each stage, the requirement specification and emerging design ideas will be shared and discussed with the SYNC system developers. Additionally, transcripts will be analysed using the TFA [10] and the Theoretical Domains Framework/Mechanisms of Action (TDF/MOA).[30-32] Where this suggests a need for behavioural support, the findings will carry forward to the EBCD.

Think-aloud technique

An ethnically-diverse group of participants who are naive to co-design focus groups and vary in confidence in using computers will be asked to think aloud as they complete tasks designed to enable them to explore key prototype functionality, observed by the researcher. Audio-recording will capture participants' comments as they conduct the tasks. Following completion of tasks, participants will be asked to complete the system usability scale (SUS), a flexible questionnaire designed to assess any technology.[33] The SUS is quick and easy to complete, consisting of 10 statements scored on a 5-point scale, with final scores ranging from 0-100 (with a SUS score above a 68 being considered above average). Audio recordings of the co-design focus groups will be transcribed verbatim and thematically analysed. Where this suggests changes to the system, these will be the basis of a prioritised list of revisions to be made. Descriptive statistics will be produced for the SUS score.

EBCD

A behavioural support intervention for patients will be developed using an EBCD approach. We will produce an animated catalyst film to stimulate co-design activities and will follow the EBCD process of showing the film to staff involved in the sHNC diagnosis pathway and patients, first separately, then jointly. The catalyst film will make use of personas and themes from the focus groups and participant quotes that exemplified themes found in the TDF/MOA informed deductive thematic analysis of focus group recordings. This is more robust than standard EBCD, in that thematic analysis is applied, rather than the informal editing process specified in EBCD. We will undertake stakeholder prioritisation exercises in the workshops, using the personas to undertake user journey mapping and pain point analysis, and then using prioritisation/ranking activities to agree on areas of focus for behavioural support intervention development. Based on focus group findings, the EVEREST-HN study team will identify prior behavioural support materials used in usual care in sHNC referral pathways. We will code the content of these materials using the Behaviour Change Technique (BCT) Taxonomy,[34] and label them according to the links to TDF/MOA constructs. Together, the catalyst film and the prior behavioural support content will provide material from which the stakeholder co-design team can together produce the novel behavioural support intervention for the pathway for sHNC referrals to secondary care, employing ideation techniques, sketching and prototyping methods. [35]

Up to four workshops with patients and staff will explore solutions to the barriers and facilitators to acceptability and engagement with the co-designed pathway, using findings from the TFA/TDF/MOA-informed deductive thematic analysis represented in the catalyst film. Co-production

For peer review only

activities will focus on adapting existing materials to bolster their acceptability, focusing firstly on the constructs, then ranking the identified prior materials for each construct on TFA, and finally adapting prior materials on these dimensions to improve their application to the pathway and acceptability to patients. Where suitable materials do not exist, these will be developed using BCT definitions linked to the identified TDF/MOA constructs as a starting point, and co-design activities will be structured with a focus on enhancing TFA constructs.

The materials will be coded for their BCT content and linked to the TDF/MOA framework by a member of the study team not involved in co-design activities. This will form the basis of a TFA/TDF/MOA-informed comparative deductive thematic analysis of the co-production activities of the workshops and the content of the intervention materials. We will operationalise findings relating to the BCT content of the finalised materials and their linked TDF/MOA constructs using the Grid Enabled Measures database[36] to identify related scales for adaptation by the study team. These will index the effects of the behavioural support intervention to support engagement with the pathway.

Patient and public involvement

We worked with three PPI groups to define the aims, objectives and design of the EVEREST-HN programme. Patients felt strongly that any new system should be as safe at detecting cancer as the current one. Patients prioritised being seen as quickly as possible when referred through the two-week wait system. Additionally, they felt that the period between referral and completing the pathway (either reassurance/discharge or diagnosis with cancer) was a very anxious time, describing a “feeling of limbo”, of being in a “vacuum”, and that the “worst part of it was the waiting”. The groups reported earlier contact, even if delivered remotely, would reduce anxiety. We have enlisted two PPI representatives to ensure that the patient perspective remains core to the EVEREST-HN programme. Both are HNC survivors. They will be active members of the project management group, contributing to the running of the project; being involved in decision-making, collaboratively pre-empting barriers, highlighting any delays against the proposed timeline and jointly generating solutions, providing their unique perspective. An ethnic diverse group of participants including people with English as a second language, will be recruited from PPI groups at the University of Bradford and their networks to co-design the SYNC symptom questionnaire.

Ethics and dissemination

This protocol has been reviewed by the Committee for Clinical Research at the Royal Marsden, a Research Ethics Committee and the Health Research Authority. Consideration of the ethical issues of working with patients on an urgent cancer diagnostic pathway has been a priority in the design of our data collection.

Our study design has been informed by our existing work with patients with HNC, including with patients shortly after diagnosis. We have found that, if the reasons for approaching people at a particular time are made clear, and the approach is made with sensitivity, patients appreciate the need for them to contacted at that time, are willing to take part, and may see participation as an opportunity for something positive to come out of a difficult situation. Our PPI representatives will continue to provide a patient perspective throughout study design and conduct. Specifically, we will work with them in operationalising our recruitment and data collection approaches.

Our PPI representatives suggested that the time prior to diagnosis is an acceptable time to conduct research for WP1, because at this point waiting can be very stressful, and having the opportunity to talk about the process is unlikely to add additional anxiety. The period around diagnosis is likely to

For peer review only

be more problematic; we wish to avoid contacting people in the period immediately following diagnosis. However, the period following this, early in the treatment process, before side effects become too challenging, is also likely to be an acceptable time to conduct qualitative interviews with patients. Any recruitment after the initial diagnostic consultation will be led by sites, who will know where patients are in their diagnosis and treatment pathway. Involvement of research nurses to obtain informed consent for participation will help to avoid additional pressure that patients might feel to participate.

Findings from the study will be submitted for publication in relevant peer reviewed journals. Reporting guidance for qualitative research be followed. We will also work with our dissemination and impact advisory group to identify other routes to dissemination for varying stakeholder groups, including patients and public, clinicians and researchers, Cancer Alliances and the technology sector. This may include material in multiple formats (presentations, social media content, infographics) to target different audiences.

For peer review only

References

1. Rogers SN, Staunton A, Girach R, et al. Audit of the two-week pathway for patients with suspected cancer of the head and neck and the influence of socioeconomic status. 2019. doi.org/ghkf6m.
2. Head and Neck Cancer patients. Patient and Public Involvement focus group for EVEREST-HN. (Teleconference). 2020.
3. Tikka, T, Kavanagh K, Lowit A, et al. Head and neck cancer risk calculator (HaNC-RC)—V.2. Adjustments and addition of symptoms and social history factors. 2020;**45**:380–388. https://doi.org/10.1111/coa.13511
4. Hardman J, Tikka T, Paleri V, et al. Remote triage incorporating symptom-based risk stratification for suspected head and neck cancer referrals: a prospective population-based study. 2022;**127**:4177-89.
5. Sud A, Torr B, Jones ME, et al. Effect of delays in the 2-week-wait cancer referral pathway during the COVID-19 pandemic on cancer survival in the UK: a modelling study. 2020;**21**:1035-44. doi.org/gg5w7k.
6. CancerData. 2020. www.cancerdata.nhs.uk. (Accessed 18th September 2023).
7. Moffat J, Hinchliffe R, Ironmonger L, et al. Identifying anticipated barriers to help-seeking to promote earlier diagnosis of cancer in Great Britain. 2016;**141**:120-5. doi.org/gjnmgw
8. Brindle L, Pope C, Corner J, et al. Eliciting symptoms interpreted as normal by patients with early-stage lung cancer: could GP elicitation of normalised symptoms reduce delay in diagnosis? Cross-sectional interview study. 2012;**2**:e001977. doi.org/gb3tk4.
9. NIHR (2020) Improving inclusion of under-served groups in clinical research: Guidance from the NIHR-INCLUDE project. UK: NIHR. Available at: www.nihr.ac.uk/documents/improving-inclusion-of-under-served-groups-in-clinical-research-guidance-from-include-project/25435 (13th September 2023)
10. Sekhon M, Cartwright M, Francis, JJ. Acceptability of healthcare interventions: an overview of reviews and development of a theoretical framework. 2017;**17**:88. https://doi.org/10.1186/s12913-017-2031-8
11. May C, Finch T. Implementing, Embedding, and Integrating Practices: An Outline of Normalization Process Theory. 2009;**43.** doi.org/dg2cbh.

12. Murray E, Treweek S, Pope C, et al. Normalisation process theory: a framework for developing, evaluating and implementing complex interventions. 2010;**8.** doi.org/bchsn9.
13. Paleri V, Patterson J, Rousseau N, et al. Gastrostomy versus nasogastric tube feeding for chemoradiation patients with head and neck cancer: the TUBE pilot RCT. 2018;**22**. doi.org/10.3310/hta22160.
14. May C, Cummings A, Girling M, et al. Using Normalization Process Theory in feasibility studies and process evaluations of complex healthcare interventions: a systematic review. 2018;**13**:80. doi.org/gdqb2p.
15. Mair F, May C, O'Donnell C, et al. Factors that promote or inhibit the implementation of e-health systems: an explanatory systematic review. 2012;**90**:357-64. doi.org/f3x8tf.
16. Bradley P, Hall N, Maniatopoulos G, et al. Factors shaping the implementation and use of Clinical Cancer Decision Tools by GPs in primary care: a qualitative framework synthesis. 2021;**11**:e043338. doi.org/gjm8j3.
17. Elwyn G, Légaré F, van der Weijden T, et al. Arduous implementation: does the Normalisation Process Model explain why it's so difficult to embed decision support technologies for patients in routine clinical practice. 2008;**3**:57. doi.org/dt8msz.
18. Ong B, Hodgson D, Small N, et al. Implementing a digital patient feedback system: an analysis using normalisation process theory. 2020;**20**;387. doi.org/gjm9bv
19. Greenhalgh T, Wherton J, Papoutsi C, et al. Beyond Adoption: A New Framework for Theorizing and Evaluating Nonadoption, Abandonment, and Challenges to the Scale-Up, Spread, and Sustainability of Health and Care Technologies. 2017; **19**:e367. doi.org/gd23jh.
20. Vindrola-Padros C, Chisnall G, Polanco N, et al. Iterative cycles in qualitative research: Introducing the RREAL Sheet as an innovative process. 2022. https://doi.org/10.31219/osf.io/9dp2w
21. Vindrola-Padros C. *Doing Rapid Qualitative Research*. Los Angeles: Sage 2021.
22. Saunders B, Sim J, Kinstone T, et al. Saturation in qualitative research: exploring its conceptualization and operationalization. 2018;**52**:1893-907. doi.org/gfgts3.
23. Rogers Y, Sharp H, Preece J. *Interaction design: beyond human-computer interaction*. 3rd ed. Chichester, West Sussex, U.K: Wiley 2011.
24. Robertson S, Robertson J. Mastering the requirements process. 2nd ed. Upper Saddle River, NJ: Addison-Wesley 2006.
25. Charters, E. The use of Think-Aloud methods in qualitative research: An introduction to Think-Aloud methods. 2003;**12**:68–82. https://doi.org/10.26522/ brocked.v12i2.38
26. Bate P, Robert G. Toward More User-Centric OD. 2007;**43**:41-66.
27. Bate P, Robert G. *Bringing user experience to healthcare improvement: the concepts, methods and practices of experience-based design*. Oxford: Radcliffe 2009.
28. Kitzinger J. *Focus Groups. Qualitative Research in Health Care*. Blackwell Publishing, Ltd; 2006.
29. Kidd PS, Parshall MB. Getting the focus and the group: enhancing analytical rigor in focus group research. 2000;**10**:293-308. doi.org/dncz46.
30. Michie S, Johnston M, Abraham C, et al. Making psychological theory useful for implementing evidence based practice: a consensus approach. 2005;**14**:26-33. doi.org/cxktr9.
31. Cane J, O'Connor D, Michie S. Validation of the theoretical domains framework for use in behaviour change and implementation research. 2012;**7**:37. doi.org/gbbhfj.
32. Carey RN, Connell LE, Johnston M. Behavior Change Techniques and Their Mechanisms of Action: A Synthesis of Links Described in Published Intervention Literature. 2019;**53**:693-707. doi.org/ggq9n7.

For peer review only

33. Holtzblatt K, Burns Wendell J, Wood S. *Rapid contextual design: a how-to guide to key techniques for user-centered design*. San Francisco: Elsevier/Morgan Kaufmann 2005.
34. Michie S, Richardson M, Johnston M, et al. The behavior change technique taxonomy (v1) of 93 hierarchically clustered techniques: building an international consensus for the reporting of behavior change interventions. 2013;**46**:81-95. doi.org/gc3jvc.
35. Grindell C, Coates E, Croot L, O'Cathain A. The use of co-production, co-design and co-creation to mobilise knowledge in the management of health conditions: a systematic review. 2022;**22**:877. doi: 10.1186/s12913-022-08079-y.
36. Moser RP, Hesse BW, Shaikh AR, et al. Grid-enabled measures: using Science 2.0 to standardize measures and share data. 2011;**40**:134-43. doi.org/c8m7kp.

For peer review only

Authors' contributions

JH, IK, JP, VP, NR, RR, Chris Elkington (CE) and John Holmes (JH) (experts by experience) conceptualised the study. NR leads WP1, RR and IK lead WP2; IK, RR and NR are joint senior authors of this manuscript. VP and JP are joint chief investigators for the EVEREST programme; JH is deputy chief investigator for the EVEREST programme. All authors and CE and JH contributed to protocol development. AA wrote the first draft of the manuscript, IK, LMcV, CO, JP, VP, NR, RR, SW reviewed and contributed to subsequent drafts. All authors reviewed and approved the final draft of the manuscript. NR, IK and RR are joint final authors.

Funding statement

This work was supported by NIHR Programme Grants for Applied Research (grant number: NIHR202862).

Competing interests

The authors declare that they have no conflict of interest.

Appendices

Appendix 1. Patient interview topic guide

EVEREST-HN

Using patient-reported symptoms to guide referral for suspected head and neck cancer

PATIENT INTERVIEW TOPIC GUIDE

Version 1.0

EVEREST-HN: Patient Interview Topic Guide

Note: The interview schedule is developmental. The questions will need to be tailored to the specific answers of each interviewee. The interview schedule given here is therefore a general topic guide for the one-to-one qualitative interviews.

Welcome and Introduction.

Ask if any questions. Obtain informed consent

Re-cap of Research and Plan for Interview

Brief re-cap on the aims and purpose of the interview and explain what will happen.

For peer review only

The following questions need not be covered in this particular order but rather the interview should flow as freely and naturally as possible. The interviewer will prompt as appropriate with phrases such as 'can you tell me a little more about that', 'can you give me an example of that', 'how did/do you feel about that'.

Experience of cancer pathway

It may be upsetting for the patient to talk about some parts of their experience. It is important to give them time to talk about this. Modify the questions about their pathway on the basis of what they say about their overall experience.

- **Can you tell me a bit about the symptoms that you have had in your head or neck and how you came to be referred for an urgent hospital appointment?**
    - Symptoms – how long, severity etc
    - Explore what they mean by terms "can you tell me a bit more about that?" "what do you mean by xxx?"
    - What prompted GP consultation (in some people it could have been picked up opportunistically at a routine dental appointment)
    - What did the GP say to you about why they were referring you to hospital?
    - How did you organise the time to go to the hospital?
        - Choice?
- **How did you feel about being referred for an urgent hospital appointment?**
    - Pleased, anxious?
- **How did you find the period while you were waiting for the hospital appointment?**
    - Anxious?
    - Information searching?
- **Can you tell me what happened at your hospital appointment?**
    - Who was there?
    - Tests etc
- **What has happened since that first hospital appointment?**
    - Tests

The EVEREST study is aiming to improve the urgent cancer referral pathway. It would be good to know from your point of view what was good about your experience and what could be improved.

- **What things were good about your experience of an urgent referral?**

For peer review only

- **What things could have been better?**

EVEREST

The next questions are about one idea to improve the urgent cancer referral pathway for patients. We would like to hear what you think of it, as someone who has been through an urgent cancer referral recently.

The idea is that patients would be asked some questions about their symptoms either on a computer or on the phone. This would be in the time between seeing the GP (or dentist) and going to the hospital. The questions might be about symptoms like a sore throat, a blocked nose or a lump in your neck. The answers would be given to the hospital doctors to look at before they see the patients. Depending on the answers they give, some people might be sent for tests straight away rather than waiting to see a doctor first.

Thinking about people like you who have an urgent referral, how do you think people would feel if they were asked to answer some questions about their symptoms on a computer or on the phone?

- Do you use a computer/smart phone?
- Do you ever use a computer or phone for things like shopping, booking tickets or checking health information (e.g. NHS 111)?
- How do you feel about using the computer for things like that?
    - Convenient or hard work/stressful?
    - Are there any websites that you can think of that are particularly bad or good?
- How do you feel about the idea of answering questions from the hospital about your health on a computer or phone?
- Do you think it would have made you feel more or less anxious to be asked to answer some questions?
- Is there anything that you think might help patients to answer the questions?
- How do you feel about the idea that a computer might help decide whether you were sent straight for tests before you saw a doctor?
    - Do you think it is ok for a computer to help with those decisions or do you think it should always be the doctor?
        - What if the computer made it quicker to get an answer about whether you have cancer or not?

For peer review only

I'd like to ask you about being invited to take part in the EVEREST-HN study. Can you tell me how you were first informed about the study?

- Who was there?
- What else was going on in the appointment?
- Can you remember what they said?
- Can you remember what your first reaction was?

How did you decide whether to take part in the study?

- How did you make your decision?
- What things were important to you?
- Did you speak to other people about the decision? If so, who?
- When did you make the decision?
  - Immediately/nurse phone call/recruitment discussion
- Did you find it an easy decision to make? Why (not)?

Do you remember being given some written information about the study?

- What did you think about that information?
- Was it helpful?
- How did you use it?
- Do you have any suggestions for how it could be improved? If so, how?

Would you have liked information about anything else?

- If so, what?
- Why was that important to you?

Anything Not Covered?

Is there anything that we haven't covered in the interview that you think we should know or think about?

Closing and Thanks

Conclude the discussion and thank the participant for their time and contribution.

For peer review only

Appendix 2. Clinician interview topic guide

EVEREST-HN

Using patient-reported symptoms to guide referral for suspected head and neck cancer

STAFF INTERVIEW TOPIC GUIDE
Version 1.0

EVEREST-HN: Staff Interview Topic Guide

Note: The interview schedule is developmental. The questions will need to be tailored to the specific answers of each interviewee. The interview schedule given here is therefore a general topic guide for the one-to-one qualitative interviews.

Welcome and Introduction.

Ask if any questions. Obtain informed consent

Re-cap of Research and Plan for Interview

Brief re-cap on the aims and purpose of the interview and explain what will happen.

For peer review only

The following questions need not be covered in this particular order but rather the interview should flow as freely and naturally as possible. The interviewer will prompt as appropriate with phrases such as 'can you tell me a little more about that', 'can you give me an example of that', 'how did/do you feel about that'.

Cancer diagnostic pathway

Initial questions about the referral pathway at the specific trust may not be needed if they have been covered in previous interviews.

Can you tell me about how the head and neck urgent cancer referral pathway is organised at the Trust?

- ***Establish in detail (who (including involvement of Nurses and AHPs), how, when):***
  - ***how referrals are processed***
  - ***how clinics are organised***
  - ***how onward referrals are made***

I'd like to know about changes that happened in the referral pathway during COVID-19 – how did you manage urgent referrals during that period?

- ***Did they use any means of triaging referrals? – if so find out about this in detail and about their experiences of this.***

Can you tell me about your role within the head and neck urgent referral cancer pathway?

The EVEREST study is aiming to improve the diagnostic pathway for urgent head and neck cancer referrals. It would be good to know from your point of view what is good about the current pathway and what could be improved.

- **What things do you think are good about the current diagnostic pathway for urgent referral?**

For peer review only

- **What things could be better?**

- **Are there particular sub-groups of patients that are more straightforward or harder to assess?**

You probably remember that we observed/recorded your consultation with (name). Would it be ok if we talked a bit about the consultation so that we can understand more about the processes that were happening?

Specific questions about e.g. why they pursued a particular line of questioning, or what triggered a decision to request a particular test

The EVEREST study is planning to introduce an intervention within the diagnostic pathway. Patients will be asked some questions about their symptoms either on a computer or on the phone. This would be triggered by the receipt of the referral at the hospital. Their answers would be used to calculate a risk score which would be made available to the hospital team, along with any additional information provided by the patient. We hope that this might enable the hospital team to make some decisions ahead of a hospital appointment, e.g. about tests or who needs to be seen most quickly and to provide relevant information in a standardised and accessible format.

What do you think of this idea?

- ***Feasible? (digital exclusion)***
- ***Useful? (an improvement? Would they trust a risk score?)***
- ***How would you see it working? (who might be involved – what roles might change? Who would need to be involved in planning for change?)***
- ***Add to or reduce workloads?***
- ***Is this the best way to improve the pathway or is there something that you think would work better?***

Anything Not Covered?

Is there anything that we haven't covered in the interview that you think we should know or think about?

Closing and Thanks

Conclude the discussion and thank the participant for their time and contribution.

For peer review only

For peer review only

BMJ Open

A qualitative study exploring the design of a patient-reported symptom-based risk stratification system for suspected head and neck cancer referrals: protocol for work packages 1 and 2 within the EVEREST-HN programme

Journal:	*BMJ Open*
Manuscript ID	bmjopen-2023-081151.R1
Article Type:	Protocol
Date Submitted by the Author:	14-Feb-2024
Complete List of Authors:	Albutt, Abigail; University of Leeds, Leeds Institute of Clinical Trials Research<br>Hardman, John; Northwick Park Hospital<br>McVey, Lynn; University of Bradford, Centre for Digital Innovations in Health & Social Care; Wolfson Centre for Applied Health Research<br>Odo, Chinasa; University of Bradford, Centre for Digital Innovations in Health & Social Care; Wolfson Centre for Applied Health Research<br>Paleri, Vinidh ; The Royal Marsden NHS Foundation Trust<br>Patterson, Jo; University of Liverpool<br>Webb, Sarah; The Royal Marsden NHS Foundation Trust<br>Rousseau, Nikki; University of Leeds, Leeds Institute of Clinical Trials Research<br>Kellar, Ian; The University of Sheffield, Department of Psychology<br>Randell, Rebecca; University of Bradford, Centre for Digital Innovations in Health & Social Care; Wolfson Centre for Applied Health Research
**Primary Subject Heading**:	Oncology
Secondary Subject Heading:	Ear, nose and throat/otolaryngology, Diagnostics, Health informatics, Health services research
Keywords:	Head & neck tumours < ONCOLOGY, Health informatics < BIOTECHNOLOGY & BIOINFORMATICS, Clinical Decision-Making, QUALITATIVE RESEARCH, Risk Factors, Surveys and Questionnaires

SCHOLARONE™
Manuscripts

A qualitative study exploring the design of a patient-reported symptom-based risk stratification system for suspected head and neck cancer referrals: protocol for work packages 1 and 2 within the EVEREST-HN programme

Authors and affiliations

1. Abigail Albutt, Clinical Trials Research Unit, Leeds Institute of Clinical Trials Research, University of Leeds, Leeds LS2 9JT, UK (A.K.Albutt@leeds.ac.uk)
2. John Hardman, Northwick Park Hospital, Watford Road, Harrow HA1 3UJ, UK (johncharles.hardman@nhs.net)
3. Lynn McVey, Centre for Digital Innovations in Health and Social Care, Faculty of Health Studies, University of Bradford, Bradford BD7 1DP, UK (l.mcvey@bradford.ac.uk)
4. Chinasa Odo, Centre for Digital Innovations in Health and Social Care, Faculty of Health Studies, University of Bradford, Bradford BD7 1DP, UK (c.r.odo@bradford.ac.uk)
5. Vinidh Paleri, Royal Marsden NHS Foundation Trust, Fulham Road, London SW3 6JJ, UK (Vinidh.Paleri@rmh.nhs.uk)
6. Joanne M Patterson, School of Health Sciences, Institute of Population Health, University of Liverpool, Liverpool L69 3GB, UK (joanne.patterson@liverpool.ac.uk)
7. Sarah Webb, Royal Marsden NHS Foundation Trust, Fulham Road, London SW3 6JJ, UK (sarah.webb2@rmh.nhs.uk)
8. Nikki Rousseau, Clinical Trials Research Unit, Leeds Institute of Clinical Trials Research, University of Leeds, Leeds LS2 9JT, UK (N.Rousseau1@leeds.ac.uk)
9. Ian Kellar, Department of Psychology, University of Sheffield, Sheffield, S1 2LT, UK (i.kellar@sheffield.ac.uk)
10. Rebecca Randell*, Centre for Digital Innovations in Health and Social Care, Faculty of Health Studies, University of Bradford, Bradford BD7 1DP, UK (r.randell@bradford.ac.uk)

*Correspondence to:
Rebecca Randell
Centre for Digital Innovations in Health and Social Care, Faculty of Health Studies, University of Bradford, Bradford BD7 1DP
r.randell@bradford.ac.uk

For peer review only

Abstract

Introduction

Between 2009/10 and 2019/20, England witnessed an increase in suspected head and neck cancer (sHNC) referrals from 140 to 404 patients per 100,000 population. One in ten patients are not seen within the two-week target, contributing to patient anxiety. We will develop a pathway for sHNC referrals, based on the Head and Neck Cancer Risk Calculator. The EVEREST-HN (evolution of a patient-reported symptom-based risk stratification system to redesign the suspected head and neck cancer referral pathway) Programme comprises six work packages. This protocol describes work packages 1 (WP1) and 2 (WP2). WP1 will obtain an understanding of language to optimise the SYmptom iNput Clinical (SYNC) system patient-reported symptom questionnaire for sHNC referrals, and outline requirements for the SYNC system. WP2 will co-design key elements of the SYNC system, including the SYNC questionnaire, and accompanying behaviour change materials.

Methods and analysis

WP1 will be conducted at three acute NHS Trusts with variation in service delivery models and ensuring a broad mixture of social, economic and cultural backgrounds of participants. Up to 150 patients with sHNC (n=50 per site) and 15 clinicians (n=5 per site) will be recruited. WP1 will use qualitative methods including interviews, observation and recordings of consultations. Rapid qualitative analysis and inductive thematic analysis will be used to analyse the data. WP2 will recruit lay patient representatives to participate in online focus groups (n=8 per focus group), think-aloud technique and experience-based co-design, and will be analysed using qualitative and quantitative approaches.

Ethics and dissemination

The Royal Marsden Committee for Clinical Research, a Research Ethics Committee (REC) and the Health Research Authority approved this protocol. All participants will give informed consent. Ethical issues of working with patients on an urgent cancer diagnostic pathway have been considered. Findings will be disseminated via journal publications, conference presentations and public engagement activities.

Keywords: diagnosis; head and neck cancer; pathway; referral; risk calculator.

Strengths and limitations of this study

- This study forms part of a large multicentre study (the EVEREST-HN programme) that will provide evidence about whether the implementation of a patient-reported symptom-based risk stratification system for sHNC referrals is safe, improves patient experience, leads to faster diagnosis and optimises healthcare resource use.
- A scoping review of the literature and data from two PhD theses were used to generate an initial list of head and neck cancer (HNC) symptoms.
- The study has substantial patient, public and other stakeholder involvement throughout all phases including patients with lived experience of HNC, members of the public without prior experience of the urgent HNC pathway and hospital staff involved in the HNC diagnostic pathway.

For peer review only

- A key strength of the study is the use of multiple methods (observation, interviews, think-aloud techniques, experience-based co-design) and theory (Theoretical Framework of Acceptability; Theoretical Domains Framework and Normalisation Process Theory) to inform the development of patient-reported symptom-based risk stratification system.
- Our approach enables an in-depth exploration from multiple perspectives – but this means sacrificing breadth - only a small number of geographical locations are included.

INTRODUCTION

In healthcare, the 'head and neck' region includes the nose, mouth, throat, voice box, thyroid and salivary glands. Many patients present to General Practitioners (GPs) with symptoms affecting these areas, including hoarse voice, throat discomfort, neck lumps, mouth ulcers and difficulty swallowing. In some patients, these symptoms may be caused by a 'head and neck cancer' (HNC), so, they will be referred to the hospital for an urgent specialist opinion. In England in 2020, 228,482 patients were referred with sHNC, making it the fifth largest group of suspected cancer referrals. After specialist assessment, the vast majority (95%) of these patients can be reassured of being cancer-free, but about 5% will be diagnosed with cancer. Standard practice in the UK is for all sHNC referrals to be offered a face-to-face consultation as their first hospital contact. Unfortunately, partly due to capacity issues, one-in-ten sHNC referrals are not seen within the two-week target.[1] Patients have reported significant anxiety in this period making any delays very undesirable.[2]

The Head and Neck Cancer Risk Calculator (HaNC-RC) symptom inventory was developed for use by specialists. The tool was developed for risk assessment to aid referral of high-risk patients to urgent specialist clinics as patients are currently seen in a chronological order.[3] HaNC-RC-v2 was deployed as a national service evaluation of sHNC referrals undergoing remote triage in secondary care during the initial peak of the coronavirus disease 2019 pandemic.[4] Clinicians were instructed to consider both the clinical history and the outcome from HaNC-RC-v2 to decide on the management plan. Clinician-led telephone triage of 4,568 referrals was recorded: 53.2% were assessed urgently; 16.4% were discharged directly and 68.7% were classed as low-risk using HaNC-RC-v2. Cancer was reported in 5.6% (n=254/4,557) and late cancers were reported in 0.4% despite urgent assessment. This work demonstrated clinicians could perform symptom-based remote triage, supported by risk stratification, medium term outcomes were acceptable and even patients undergoing urgent assessment could have missed cancers.[4] The rapidity of progression of HNC is demonstrated by the impact delays to treatment have on outcomes. It is estimated a one-month delay to treatment led to 437, 514, and 1,415 life years lost for oropharynx, larynx and oral cavity cancers respectively.[5] With increasing numbers of referrals, an improved pathway that identifies people at higher risk for rapid assessment could offer significant benefits.[6]

The EVEREST-HN programme aims to develop and evaluate a patient-reported symptom-based risk stratification system as part of a new pathway for sHNC referrals (See Figure 1 for a study flowchart of standard care and EVEREST-HN pathways). Patients (with help from family/carers where needed), will be asked to complete an electronic questionnaire about their symptoms soon after referral. Building on our previous work, we will develop a risk stratification system for HNC based on thousands of new referrals. Hospital staff will review the information from the symptom questionnaire and the patient's individualised risk score to advise the most appropriate management before the patient comes to hospital. Obtaining symptom information from patients before they attend hospital, rather than waiting until their urgent specialist appointment, allows for earlier risk stratification. Using this system, higher-risk patients may have targeted investigations arranged directly, before being seen in clinic, and lower-risk patients may avoid unnecessary investigations before being reassured.

For peer review only

Patients vary in their confidence in describing their symptoms[7] and so, when taking the patient history, clinicians use accessible and easily interpreted questions. Therefore, it is important the questions used to elicit patient-reported symptoms are understandable to patients. Development of the EVEREST-HN pathway must be based on a comprehensive understanding of existing HNC diagnostic pathways and what patients and clinicians value. Work package 1 (WP1) aims to understand: how clinicians ask questions and decide subsequent steps for patients referred with sHNC; the language patients and clinicians use to describe symptoms;[8] how clinicians reassure and discharge low-risk patients; patients' and clinicians' views of the current diagnostic process for HNC. Work package 2 (WP2) aims to co-design key elements of the SYmptom iNput Clinical (SYNC) system, including the SYNC symptom questionnaire and behaviour change intervention materials to integrate the system into existing hospital workflows. Table 1 describes the subsequent work packages (3-6) of the EVEREST-HN programme and demonstrates how WP1 and WP2 integrate into the wider programme of work. Figure 2 shows an overview of how the work packages interlink.

Table 1. Brief description of EVEREST-HN programme work packages (WP) 3–6

**Work package**	**Brief description**
3: Informatics linkage of national datasets for suspected HNC referrals	To use data from routinely collected electronic health records to describe the sHNC population and their diagnostic pathway, and provide prognostic information for WP4 and 6.
4: SYNC system development phase-3: Feasibility study to finalise EVEREST-HN pathway	To conduct a concurrent data collection exercise and feasibility study, using the SYNC symptom questionnaire developed in WP1 and 2, alongside the prognostic data from WP3, to develop the SYNC risk stratification algorithm, agree management thresholds and recommendations by stakeholder consensus, finalise all elements of the EVEREST-HN pathway.
5: Cluster randomised controlled trial	To conduct a cluster randomised-trial, with 6-month pilot, to compare the new pathway to standard care, using a primary outcome of cancer diagnosed within 62 days.
6: Health economics	To evaluate the cost-effectiveness of the pathway.

METHODS AND ANALYSIS

<u>WP1: Qualitative research to generate SYNC symptom questionnaire</u>

Study design

WP1 will employ qualitative methods including interviews, observation and recordings of consultations, and analysis of referral documentation. Work will be conducted at three sites with variation in service delivery models, including those not traditionally involved in research. Sites will be recruited to ensure a broad mixture of social, economic and cultural backgrounds of potential participants. Approximately 150 adults (n=50 per site) referred for sHNC, and 15 clinicians (n= 5 per site including different subspecialties that see sHNC patients), will be recruited. A subset of recruited

For peer review only

patients (approx n=30) will be interviewed. Clinicians (n=15 varying grades and specialty) will be interviewed and interviews may include a review of extracts of recordings of their consultations to understand actions taken within and as a result of the consultation. Data collection started in April 2023 and is due to be completed in April 2024.

Sample

Sites will be chosen for variation in service delivery model and to facilitate diversity in patient characteristics. Patient recruitment will aim for variation in age, ethnicity, socio-economic status, presenting symptoms. Clinicians recruited will be clinical staff involved in HNC diagnosis, varying in seniority (consultant, registrar) and specialty (ear, nose and throat surgery, oral and maxillofacial surgery). Recruitment is not restricted to English speakers. Where a consultation is conducted with the support of an interpretation service or is conducted in a language other than English (where recruited clinicians speak the language of the patient), these patients are eligible for inclusion in the study. Study materials are available in the most common non-English languages at each site to facilitate this.

Our target sample sizes have been selected to balance breadth and depth. We aim to achieve variety in terms of participant characteristics but also an in-depth understanding of how clinicians vary in their approach to the diagnostic consultation. Our target sample size of 150 has been chosen to enable sufficient patients per clinician (approximately 10 patients per clinician) to identify variation in approach to the diagnostic consultation, and also variation in clinical (particularly HaNC-RC criteria) and socio-demographic characteristics of patients at each site. An initial phase of recruitment will involve recordings of one or two clinics per participating clinician at each site (n=approximately 7 consenting patients per clinician, total n=105). Subsequent recruitment (estimate additional n=45) will target patients referred with symptoms or characteristics not well represented in the initial sample (referral letters will be reviewed by hospital staff to identify potential patients). Recruitment will be monitored frequently throughout data collection/analysis to ensure that the range of characteristics and numbers of participants are adequate to enable our research questions to be addressed. Recruitment will cease when we have "adequate data to tell a rich, complex and multi-faceted storey about patternings related to the phenomena of interest"[9] The aim of our rapid qualitative analysis is to provide an understanding of the language used and key requirements for the SYNC system, not necessarily to generate themes or a theory. We have previously referred to this point in rapid qualitative analysis as "analytic saturation"[10].

Inclusion criteria
Patients: adults ≥ 18 years referred via sHNC pathway without previous history of HNC. Participant is willing and able to give informed consent for participation in the study.

Clinicians: Staff at participating sites involved in the diagnostic pathway for people with sHNC.

Exclusion criteria
Individuals who do not meet the inclusion criteria.

Methodology

Consent
Members of the local clinical team will proactively identify eligible patients through triage of referral letters. Eligible patients will be those allocated to attend a diagnostic clinic, or with symptom(s) or other characteristics identified as being important to include in the sample. Where possible, the Patient Information Sheet will be sent to patients with appointment details.

For peer review only

Participants will be approached and consented by local care teams, who will also confirm eligibility. Written consent for consultations to be recorded will be taken immediately prior to appointments. Where patients are willing, consent will also be taken for their contact details to be shared with the research team, so they can potentially be invited to participate in an interview. Verbal consent will be taken and recorded prior to interviews conducted remotely.

The EVEREST-HN qualitative researchers will conduct a small number of clinic observations and qualitative research with participants who have consented to be involved. For clinic observations, informed consent will be received for both clinician and patient participants.

Data collection

Data collection will comprise:

- Non-participant observation and audio-recordings of diagnostic consultations.
- Review of referral documentation
- Patient interviews
    - the focus will be on their experience of the current diagnostic pathway - we will also seek their views on the EVEREST-HN pathway.
- Clinician interviews.
    - the focus will be on the clinical decision-making process, including views on the EVEREST-HN pathway.

Our recruitment strategy has been co-developed with Patient and Public Involvement (PPI) and with reference to NIHR INCLUDE guidance[11]. Recruitment information will be available in different formats and languages. Topic guides for the interviews will be informed by our own and other research, our PPI and by our theoretical framework including Theoretical Framework of Acceptability (TFA)[12] and Normalisation Process Theory (NPT)[13]which can help to sensitise researchers to relevant aspects of context.[14] See Appendix 1 and Appendix 2 for patient and clinician interview topic guides, respectively.

Dataset and case report form (CRF)

Limited descriptive information will be captured to monitor included participants' characteristics, consider sub-groups in the qualitative analysis, and describe participants in study outputs.

The following fields will be recorded on the CRF:

· Demographics (age, gender, ethnicity)
· Referral criteria (e.g., throat lump - based on standard referral forms)
· Smoking status
· Outcome of initial consultation (discharge, further tests, referral to other service)
· Diagnostic outcome (HNC diagnosed, other/no cancer diagnosis)

Pseudonymisation

Sites will generate a unique study ID for each participant and use a 'key' to reference this to the NHS and hospital medical record number. This key will be stored locally at contributing trusts on an excel file on the hard drive of a secure NHS computer. The study key will be stored for the duration of the study and then destroyed in line with local processes for handling patient identifiable data.
Study ID will comprise a single letter hospital code followed by a three-digit consecutive number, e.g. A001, A002, etc.

Theoretical framework

For peer review only

Data collection and analysis will be informed by our theoretical framework which includes TFA and NPT.[12,13] TFA consists of seven constructs: affective attitude; burden; ethicality; intervention coherence; opportunity costs; perceived effectiveness; and self-efficacy.[12] It can be used to highlight characteristics of healthcare interventions that could be improved. NPT considers factors that affect implementation in four areas: coherence; cognitive participation; collective action; reflexive monitoring.[13] NPT has been widely used in studies on implementation of interventions in health care including in trial process evaluations,[15,16] e-health[17] and decision aids.[18,19] In e-health its use has highlighted the importance of sense-making (users' understanding of the e-health intervention's purpose and its differences from usual practice), the impact of digital interventions on roles and responsibilities and the work that must be done to incorporate e-health interventions into pathways.[17,20] Our theoretical framework will be reviewed regularly throughout WP1 and WP2. We will consider whether the existing framework fits the data collected, and whether additional theories, such as the non-adoption, abandonment, scale-up, spread, and sustainability (NASS) framework[21] would be helpful.

Data storage

Study database
Descriptive information about patients and their health will be entered by site staff into MACRO, a secure database, for transfer to and storage at the University of Leeds. Participants will be identified by their unique study ID.

Qualitative data will be translated to English (where required) and transcribed by an approved third party before being anonymised. The original recordings will be stored securely separately from transcripts and retained for a minimum of 5 years after project-end for data verification, if required. Contact information will be stored until it is no longer needed to contact participants.

Anonymised transcripts and associated pseudonymised descriptive information about participants will be retained after the end of the study to enable data sharing. It will be possible to link the transcript to the descriptive information via the study ID, as this is likely to be essential information as part of data sharing, but after the contact information has been deleted it will not be possible for anyone to link the anonymised information to any identifiable information.

Data analysis
All interviews will be audio-recorded and transcribed verbatim. Both transcripts and contemporaneous field notes from non-participant observation in clinical settings will be edited to ensure participant anonymity. Analysis will proceed concurrently with data collection using a rapid qualitative analysis approach and will be conducted according to standard procedures of rigorous rapid qualitative analysis [22,23]. Separate Rapid Assessment Procedure (RAP) sheets[22] will be developed for patient interviews, clinician interviews, and consultations based on the research questions. Researchers will independently complete RAP sheets after each interview and for each recorded consultation. We will hold 'data clinics' where the research team exchanges interpretations of key issues emerging from the data. These will facilitate decisions regarding analytic saturation.[10, 24]

Content analysis of consultation recordings will focus on language used, generating a glossary of terms used by patients to describe their symptoms, and aiming to identify language used by clinicians which is helpful in generating relevant responses from patients, as well as terms that seem to be poorly understood. Thematic analysis of interviews will inform guiding principles (intervention objectives and key design features) and be used to develop a requirement specification and "personas" for co-design. The requirement specification will detail both functional requirements

For peer review only

(what the SYNC system should do) and non-functional requirements (including look and feel, usability, performance, and maintainability and support requirements, and requirements relating to implementation).[25] The requirements will be presented using the Volere format.[26]

WP2: Co-design of the SYNC symptom questionnaire

Study design
The co-design process will be iterative, and will involve a series of interactive focus groups, think-aloud technique[27] and experience-based co-design (EBCD).[28]

Sample
An ethnic diverse group including participants with English as a second language, will be recruited from PPI groups at the University of Bradford and their networks. We will also involve the EVEREST-HN PPI representatives, to provide their experience of the HNC pathway. Based on previous experience of co-design, we aim to have eight participants.

Methodology

Data collection and analysis

Focus groups

These will be undertaken via Microsoft Teams and recorded. Each focus group will last approximately 90 minutes. Co-design requires careful preparation but also flexibility, to allow participant involvement in the design of the approach and the intervention.[29] While open to revision and further specification, we will move from discussing personas and gathering participants' perspectives on what the questionnaire should look like, to discussion of a paper-based prototype to encourage further design input, and discussion of a high fidelity prototype, in terms of how comprehensible, usable, and acceptable it is. Throughout the focus groups the moderator will encourage discussion of differences of opinions.[30] At the end of each focus group, the moderator will present any tentatively identified issues to participants for confirmation or clarification.[31] Participants will receive incentives for their time and contribution toward the co-design process.

Audio recordings will be transcribed verbatim. Data analysis will be conducted after each focus group using thematic analysis and will contribute towards refinements to the requirement specification and determine priorities for subsequent prototypes. At each stage, the requirement specification and emerging design ideas will be shared and discussed with the SYNC system developers. Additionally, transcripts will be analysed using the TFA [12] and the Theoretical Domains Framework/Mechanisms of Action (TDF/MOA).[32-34] Where this suggests a need for behavioural support, the findings will carry forward to the EBCD.

Think-aloud technique
An ethnically-diverse group of participants who are naive to co-design focus groups and vary in confidence in using computers will be asked to think aloud as they complete tasks designed to enable them to explore key prototype functionality, observed by the researcher. Audio-recording will capture participants' comments as they conduct the tasks. Following completion of tasks, participants will be asked to complete the system usability scale (SUS), a flexible questionnaire designed to assess any technology.[35] The SUS is quick and easy to complete, consisting of 10 statements scored on a 5-point scale, with final scores ranging from 0-100 (with a SUS score above a 68 being considered above average). Audio recordings of the co-design focus groups will be transcribed verbatim and thematically analysed. Where this suggests changes to the system, these

For peer review only - http://bmjopen.bmj.com/site/about/guidelines.xhtml

will be the basis of a prioritised list of revisions to be made. Descriptive statistics will be produced for the SUS score.

EBCD

A behavioural support intervention for patients will be developed using an EBCD approach. We will produce an animated catalyst film to stimulate co-design activities and will follow the EBCD process of showing the film to staff involved in the sHNC diagnosis pathway and patients, first separately, then jointly. The catalyst film will make use of personas and themes from the focus groups and participant quotes that exemplified themes found in the TDF/MOA informed deductive thematic analysis of focus group recordings. This is more robust than standard EBCD, in that thematic analysis is applied, rather than the informal editing process specified in EBCD. We will undertake stakeholder prioritisation exercises in the workshops, using the personas to undertake user journey mapping and pain point analysis, and then using prioritisation/ranking activities to agree on areas of focus for behavioural support intervention development. Based on focus group findings, the EVEREST-HN study team will identify prior behavioural support materials used in usual care in sHNC referral pathways. We will code the content of these materials using the Behaviour Change Technique (BCT) Taxonomy,[34,36] and label them according to the links to TDF/MOA constructs. Together, the catalyst film and the prior behavioural support content will provide material from which the stakeholder co-design team can together produce the novel behavioural support intervention for the pathway for sHNC referrals to secondary care, employing ideation techniques, sketching and prototyping methods.[37]

Up to four workshops with patients and staff will explore solutions to the barriers and facilitators to acceptability and engagement with the co-designed pathway, using findings from the TFA/TDF/MOA-informed deductive thematic analysis represented in the catalyst film. Co-production activities will focus on adapting existing materials to bolster their acceptability, focusing firstly on the constructs, then ranking the identified prior materials for each construct on TFA, and finally adapting prior materials on these dimensions to improve their application to the pathway and acceptability to patients. Where suitable materials do not exist, these will be developed using BCT definitions linked to the identified TDF/MOA constructs as a starting point, and co-design activities will be structured with a focus on enhancing TFA constructs.

The materials will be coded for their BCT content and linked to the TDF/MOA framework by a member of the study team not involved in co-design activities. This will form the basis of a TFA/TDF/MOA-informed comparative deductive thematic analysis of the co-production activities of the workshops and the content of the intervention materials. We will operationalise findings relating to the BCT content of the finalised materials and their linked TDF/MOA constructs using the Grid Enabled Measures database[38] to identify related scales for adaptation by the study team. These will index the effects of the behavioural support intervention to support engagement with the pathway.

Patient and public involvement

We worked with three PPI groups to define the aims, objectives and design of the EVEREST-HN programme. Patients felt strongly that any new system should be as safe at detecting cancer as the current one. Patients prioritised being seen as quickly as possible when referred through the two-week wait system. Additionally, they felt that the period between referral and completing the pathway (either reassurance/discharge or diagnosis with cancer) was a very anxious time, describing a “feeling of limbo”, of being in a “vacuum”, and that the “worst part of it was the waiting”. The groups reported earlier contact, even if delivered remotely, would reduce anxiety. We have enlisted two PPI representatives to ensure that the patient perspective remains core to the EVEREST-HN

For peer review only

programme. Both are HNC survivors. They will be active members of the project management group, contributing to the running of the project; being involved in decision-making, collaboratively pre-empting barriers, highlighting any delays against the proposed timeline and jointly generating solutions, providing their unique perspective. An ethnically diverse group of participants, including people with English as a second language, will be recruited from PPI groups at the University of Bradford and their networks to co-design the SYNC symptom questionnaire.

ETHICS AND DISSEMINATION

This protocol has been reviewed and approved by the Committee for Clinical Research at the Royal Marsden (Reference: CCR5683), a Research Ethics Committee and the Health Research Authority (REC reference: 22/NW/0327). All participants will give fully informed consent to take part in the study. Consideration of the ethical issues of working with patients on an urgent cancer diagnostic pathway has been a priority in the design of our data collection.

Our study design has been informed by our existing work with patients with HNC, including with patients shortly after diagnosis. We have found that, if the reasons for approaching people at a particular time are made clear, and the approach is made with sensitivity, patients appreciate the need for them to contacted at that time, are willing to take part, and may see participation as an opportunity for something positive to come out of a difficult situation. Our PPI representatives will continue to provide a patient perspective throughout study design and conduct. Specifically, we will work with them in operationalising our recruitment and data collection approaches.

Our PPI representatives suggested that the time prior to diagnosis is an acceptable time to conduct research for WP1, because at this point waiting can be very stressful, and having the opportunity to talk about the process is unlikely to add additional anxiety. The period around diagnosis is likely to be more problematic; we wish to avoid contacting people in the period immediately following diagnosis. However, the period following this, early in the treatment process, before side effects become too challenging, is also likely to be an acceptable time to conduct qualitative interviews with patients. Any recruitment after the initial diagnostic consultation will be led by sites, who will know where patients are in their diagnosis and treatment pathway. Involvement of research nurses to obtain informed consent for participation will help to avoid additional pressure that patients might feel to participate.

In terms of risks to recruitment, poor recruitment either of patients in general or patients in specific groups could lead to an inadequate data set or data which are not reflective of the diversity of patients. Slow recruitment also risks delaying subsequent work packages. Our PPI is key to developing a recruitment strategy which is engaging and appropriate. Our experts by experience will also assist us with trouble shooting in the event of slower than anticipated recruitment. Within the EVEREST-HN programme there is planned resource to allow for involvement of research nurses and ear nose and throat trainees at sites to support site and recruitment activity. We will use various methods to contact potential patient participants for interview including telephone and email, in line with their preferred contact method. Shared working across WP1 and WP2 will ensure timely sharing of interim findings - mitigating the risk of delays impacting on onward work packages.

Findings from the study will be submitted for publication in relevant peer reviewed journals. Reporting guidance for qualitative research will be followed. We will also work with our dissemination and impact advisory group to identify other routes to dissemination for varying stakeholder groups, including patients and public, clinicians and researchers, Cancer Alliances and the technology sector. This may include material in multiple formats (presentations, social media content, infographics) to target different audiences.

For peer review only

Contributors

JH, IK, JP, VP, NR, RR conceptualised the study. NR leads WP1, RR and IK lead WP2; IK, RR and NR are joint senior authors of this manuscript. VP and JP are joint chief investigators for the EVEREST programme; JH is deputy chief investigator for the EVEREST programme. All authors contributed to protocol development. AA wrote the first draft of the manuscript, IK, LMcV, CO, JP, VP, NR, RR, SW reviewed and contributed to subsequent drafts. All authors reviewed and approved the final draft of the manuscript. NR, IK and RR are joint final authors.

Acknowledgements

Thank you to Chris Elkington and John Holmes (experts by experience) for their involvement in conceptualising the study and contributing to protocol development.

Funding

This work was supported by NIHR Programme Grants for Applied Research (grant number: NIHR202862).

Competing interests

The authors declare that they have no conflict of interest.

References

1. Rogers SN, Staunton A, Girach R, et al. Audit of the two-week pathway for patients with suspected cancer of the head and neck and the influence of socioeconomic status. 2019. doi.org/ghkf6m.
2. Head and Neck Cancer patients. Patient and Public Involvement focus group for EVEREST-HN. (Teleconference). 2020.
3. Tikka, T, Kavanagh K, Lowit A, et al. Head and neck cancer risk calculator (HaNC-RC)—V.2. Adjustments and addition of symptoms and social history factors. 2020;**45**:380–388. https://doi.org/10.1111/coa.13511
4. Hardman J, Tikka T, Paleri V, et al. Remote triage incorporating symptom-based risk stratification for suspected head and neck cancer referrals: a prospective population-based study. 2022;**127**:4177-89.
5. Sud A, Torr B, Jones ME, et al. Effect of delays in the 2-week-wait cancer referral pathway during the COVID-19 pandemic on cancer survival in the UK: a modelling study. 2020;**21**:1035-44. doi.org/gg5w7k.
6. CancerData. 2020. www.cancerdata.nhs.uk. (Accessed 18th September 2023).
7. Moffat J, Hinchliffe R, Ironmonger L, et al. Identifying anticipated barriers to help-seeking to promote earlier diagnosis of cancer in Great Britain. 2016;**141**:120-5. doi.org/gjnmgw
8. Brindle L, Pope C, Corner J, et al. Eliciting symptoms interpreted as normal by patients with early-stage lung cancer: could GP elicitation of normalised symptoms reduce delay in diagnosis? Cross-sectional interview study. 2012;**2**:e001977. doi.org/gb3tk4.
9. Braun V, and Clarke V. To saturate or not to saturate? Questioning data saturation as a useful concept for thematic analysis and sample-size rationales. 2021;**13**:201–216. doi: 10.1080/2159676X.2019.1704846

For peer review only

10. Higham R, Pini S, Quyn A, et al. Rapid qualitative analysis in a mixed-methods evaluation of an infection prevention intervention in a UK hospital setting during the COVID-19 pandemic: A discussion of the CLEAN study methodology. 2022;7. 958250. ISSN 2297-7775
11. NIHR (2020) Improving inclusion of under-served groups in clinical research: Guidance from the NIHR-INCLUDE project. UK: NIHR. Available at: www.nihr.ac.uk/documents/improving-inclusion-of-under-served-groups-in-clinical-research-guidance-from-include-project/25435 (13th September 2023)
12. Sekhon M, Cartwright M, Francis, JJ. Acceptability of healthcare interventions: an overview of reviews and development of a theoretical framework. 2017;**17**:88. https://doi.org/10.1186/s12913-017-2031-8
13. May C, Finch T. Implementing, Embedding, and Integrating Practices: An Outline of Normalization Process Theory. 2009;**43.** doi.org/dg2cbh.
14. Murray E, Treweek S, Pope C, et al. Normalisation process theory: a framework for developing, evaluating and implementing complex interventions. 2010;**8.** doi.org/bchsn9.
15. Paleri V, Patterson J, Rousseau N, et al. Gastrostomy versus nasogastric tube feeding for chemoradiation patients with head and neck cancer: the TUBE pilot RCT. 2018;**22**. doi.org/10.3310/hta22160.
16. May C, Cummings A, Girling M, et al. Using Normalization Process Theory in feasibility studies and process evaluations of complex healthcare interventions: a systematic review. 2018;**13**:80. doi.org/gdqb2p.
17. Mair F, May C, O'Donnell C, et al. Factors that promote or inhibit the implementation of e-health systems: an explanatory systematic review. 2012;**90**:357-64. doi.org/f3x8tf.
18. Bradley P, Hall N, Maniatopoulos G, et al. Factors shaping the implementation and use of Clinical Cancer Decision Tools by GPs in primary care: a qualitative framework synthesis. 2021;**11**:e043338. doi.org/gjm8j3.
19. Elwyn G, Légaré F, van der Weijden T, et al. Arduous implementation: does the Normalisation Process Model explain why it's so difficult to embed decision support technologies for patients in routine clinical practice. 2008;**3**:57. doi.org/dt8msz.
20. Ong B, Hodgson D, Small N, et al. Implementing a digital patient feedback system: an analysis using normalisation process theory. 2020;**20**;387. doi.org/gjm9bv
21. Greenhalgh T, Wherton J, Papoutsi C, et al. Beyond Adoption: A New Framework for Theorizing and Evaluating Nonadoption, Abandonment, and Challenges to the Scale-Up, Spread, and Sustainability of Health and Care Technologies. 2017; **19**:e367. doi.org/gd23jh.
22. Vindrola-Padros C, Chisnall G, Polanco N, et al. Iterative cycles in qualitative research: Introducing the RREAL Sheet as an innovative process. 2022. https://doi.org/10.31219/osf.io/9dp2w
23. Vindrola-Padros C. *Doing Rapid Qualitative Research*. Los Angeles: Sage 2021.
24. Saunders B, Sim J, Kinstone T, et al. Saturation in qualitative research: exploring its conceptualization and operationalization. 2018;**52**:1893-907. doi.org/gfgts3.
25. Rogers Y, Sharp H, Preece J. *Interaction design: beyond human-computer interaction*. 3rd ed. Chichester, West Sussex, U.K: Wiley 2011.
26. Robertson S, Robertson J. Mastering the requirements process. 2nd ed. Upper Saddle River, NJ: Addison-Wesley 2006.
27. Charters, E. The use of Think-Aloud methods in qualitative research: An introduction to Think-Aloud methods. 2003;**12**:68–82. https://doi.org/10.26522/ brocked.v12i2.38
28. Bate P, Robert G. Toward More User-Centric OD. 2007;**43**:41-66.
29. Bate P, Robert G. *Bringing user experience to healthcare improvement: the concepts, methods and practices of experience-based design*. Oxford: Radcliffe 2009.

For peer review only

30. Kitzinger J. *Focus Groups. Qualitative Research in Health Care*. Blackwell Publishing, Ltd; 2006.
31. Kidd PS, Parshall MB. Getting the focus and the group: enhancing analytical rigor in focus group research. 2000;**10**:293-308. doi.org/dncz46.
32. Michie S, Johnston M, Abraham C, et al. Making psychological theory useful for implementing evidence based practice: a consensus approach. 2005;**14**:26-33. doi.org/cxktr9.
33. Cane J, O'Connor D, Michie S. Validation of the theoretical domains framework for use in behaviour change and implementation research. 2012;**7**:37. doi.org/gbbhfj.
34. Carey RN, Connell LE, Johnston M. Behavior Change Techniques and Their Mechanisms of Action: A Synthesis of Links Described in Published Intervention Literature. 2019;**53**:693-707. doi.org/ggq9n7.
35. Holtzblatt K, Burns Wendell J, Wood S. *Rapid contextual design: a how-to guide to key techniques for user-centered design*. San Francisco: Elsevier/Morgan Kaufmann 2005.
36. Michie S, Richardson M, Johnston M, et al. The behavior change technique taxonomy (v1) of 93 hierarchically clustered techniques: building an international consensus for the reporting of behavior change interventions. 2013;**46**:81-95. doi.org/gc3jvc.
37. Grindell C, Coates E, Croot L, O'Cathain A. The use of co-production, co-design and co-creation to mobilise knowledge in the management of health conditions: a systematic review. 2022;**22**:877. doi: 10.1186/s12913-022-08079-y.
38. Moser RP, Hesse BW, Shaikh AR, et al. Grid-enabled measures: using Science 2.0 to standardize measures and share data. 2011;**40**:134-43. doi.org/c8m7kp.

Figure legends

Figure 1. Study flowchart showing standard care and EVEREST-HN pathways

Figure 2. Overview of EVERST-HN work packages

For peer review only

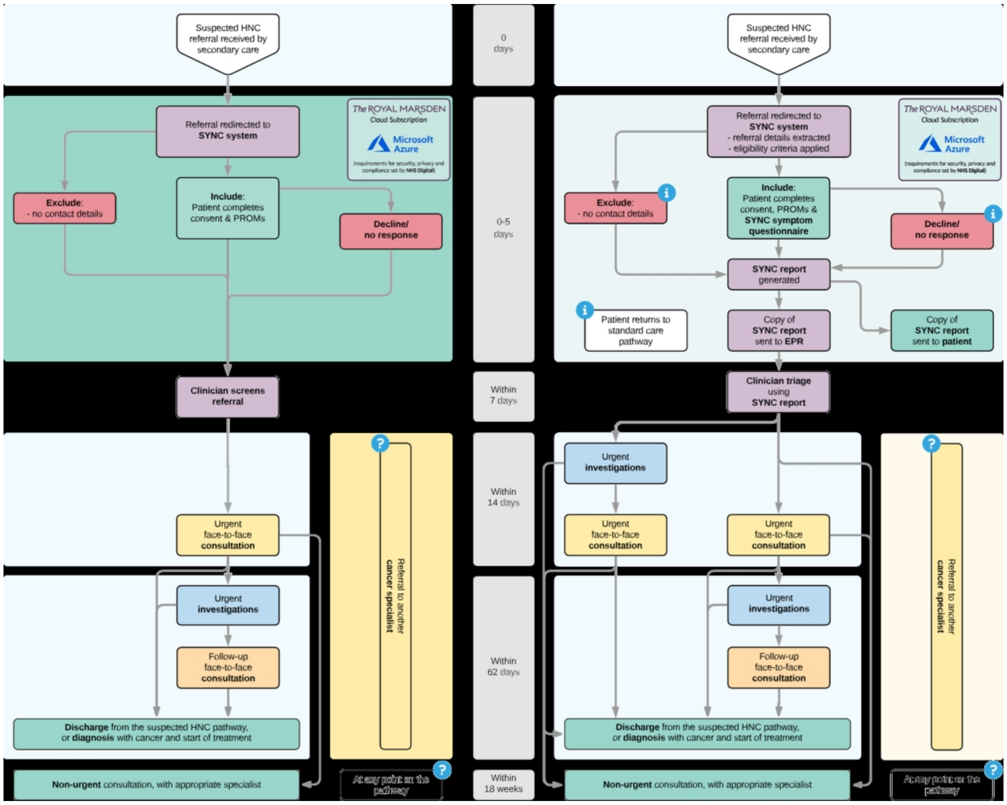

Figure 1. Study Flowchart showing standard care and EVEREST-HN pathways

215x171mm (300 x 300 DPI)

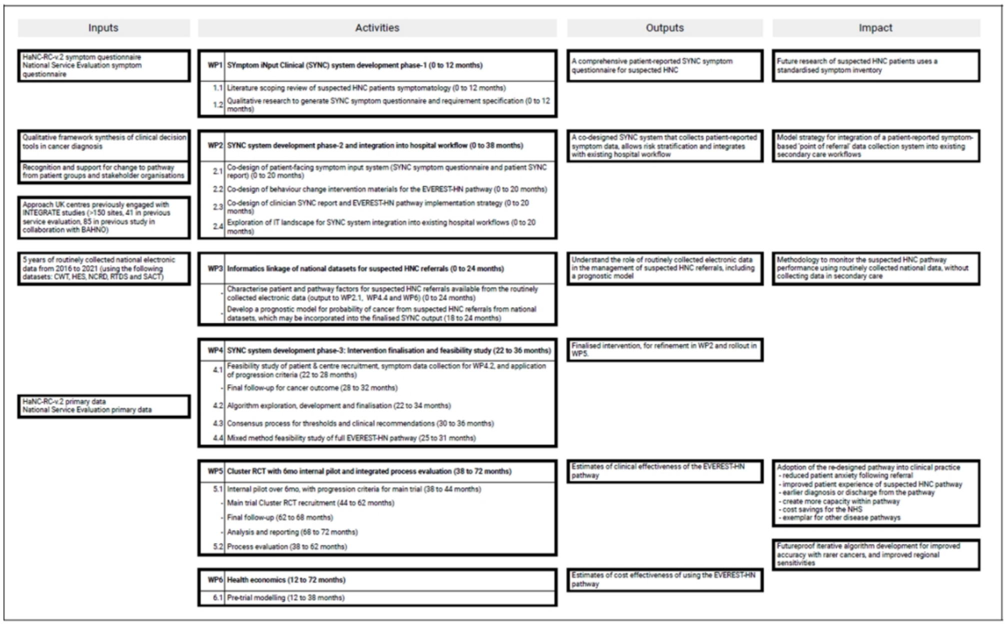

Figure 2. Overview of EVEREST-HN work packages

226x140mm (144 x 144 DPI)

Appendix 1. Patient interview topic guide

EVEREST-HN

Using patient-reported symptoms to guide referral for suspected head and neck cancer

PATIENT INTERVIEW TOPIC GUIDE

Version 1.0

For peer review only

EVEREST-HN: Patient Interview Topic Guide

Note: The interview schedule is developmental. The questions will need to be tailored to the specific answers of each interviewee. The interview schedule given here is therefore a general topic guide for the one-to-one qualitative interviews.

Welcome and Introduction.

Ask if any questions. Obtain informed consent

Re-cap of Research and Plan for Interview

Brief re-cap on the aims and purpose of the interview and explain what will happen.

The following questions need not be covered in this particular order but rather the interview should flow as freely and naturally as possible. The interviewer will prompt as appropriate with phrases such as 'can you tell me a little more about that', 'can you give me an example of that', 'how did/do you feel about that'.

Experience of cancer pathway

It may be upsetting for the patient to talk about some parts of their experience. It is important to give them time to talk about this. Modify the questions about their pathway on the basis of what they say about their overall experience.

- **Can you tell me a bit about the symptoms that you have had in your head or neck and how you came to be referred for an urgent hospital appointment?**
    - Symptoms – how long, severity etc
    - Explore what they mean by terms "can you tell me a bit more about that?" "what do you mean by xxx?"
    - What prompted GP consultation (in some people it could have been picked up opportunistically at a routine dental appointment)
    - What did the GP say to you about why they were referring you to hospital?
    - How did you organise the time to go to the hospital?
        - Choice?
- **How did you feel about being referred for an urgent hospital appointment?**
    - Pleased, anxious?
- **How did you find the period while you were waiting for the hospital appointment?**
    - Anxious?
    - Information searching?
- **Can you tell me what happened at your hospital appointment?**
    - Who was there?
    - Tests etc
- **What has happened since that first hospital appointment?**
    - Tests

The EVEREST study is aiming to improve the urgent cancer referral pathway. It would be good to know from your point of view what was good about your experience and what could be improved.

- **What things were good about your experience of an urgent referral?**

- **What things could have been better?**

EVEREST

The next questions are about one idea to improve the urgent cancer referral pathway for patients. We would like to hear what you think of it, as someone who has been through an urgent cancer referral recently.

The idea is that patients would be asked some questions about their symptoms either on a computer or on the phone. This would be in the time between seeing

For peer review only

the GP (or dentist) and going to the hospital. The questions might be about symptoms like a sore throat, a blocked nose or a lump in your neck. The answers would be given to the hospital doctors to look at before they see the patients. Depending on the answers they give, some people might be sent for tests straight away rather than waiting to see a doctor first.

Thinking about people like you who have an urgent referral, how do you think people would feel if they were asked to answer some questions about their symptoms on a computer or on the phone?

- Do you use a computer/smart phone?
- Do you ever use a computer or phone for things like shopping, booking tickets or checking health information (e.g. NHS 111)?
- How do you feel about using the computer for things like that?
    - Convenient or hard work/stressful?
    - Are there any websites that you can think of that are particularly bad or good?
- How do you feel about the idea of answering questions from the hospital about your health on a computer or phone?
- Do you think it would have made you feel more or less anxious to be asked to answer some questions?
- Is there anything that you think might help patients to answer the questions?
- How do you feel about the idea that a computer might help decide whether you were sent straight for tests before you saw a doctor?
    - Do you think it is ok for a computer to help with those decisions or do you think it should always be the doctor?
        - What if the computer made it quicker to get an answer about whether you have cancer or not?

I'd like to ask you about being invited to take part in the EVEREST-HN study. Can you tell me how you were first informed about the study?

- Who was there?
- What else was going on in the appointment?
- Can you remember what they said?
- Can you remember what your first reaction was?

How did you decide whether to take part in the study?

- How did you make your decision?
- What things were important to you?
- Did you speak to other people about the decision? If so, who?
- When did you make the decision?
    - Immediately/nurse phone call/recruitment discussion
- Did you find it an easy decision to make? Why (not)?

For peer review only

Do you remember being given some written information about the study?

- What did you think about that information?
- Was it helpful?
- How did you use it?
- Do you have any suggestions for how it could be improved? If so, how?

Would you have liked information about anything else?

- If so, what?
- Why was that important to you?

Anything Not Covered?

Is there anything that we haven't covered in the interview that you think we should know or think about?

Closing and Thanks

Conclude the discussion and thank the participant for their time and contribution.

For peer review only

Appendix 2. Clinician interview topic guide

EVEREST-HN

Using patient-reported symptoms to guide referral for suspected head and neck cancer

STAFF INTERVIEW TOPIC GUIDE

Version 1.0

EVEREST-HN: Staff Interview Topic Guide

Note: The interview schedule is developmental. The questions will need to be tailored to the specific answers of each interviewee. The interview schedule given here is therefore a general topic guide for the one-to-one qualitative interviews.

Welcome and Introduction.

Ask if any questions. Obtain informed consent

Re-cap of Research and Plan for Interview

Brief re-cap on the aims and purpose of the interview and explain what will happen.

The following questions need not be covered in this particular order but rather the interview should flow as freely and naturally as possible. The interviewer will prompt as appropriate with phrases such as 'can you tell me a little more about that', 'can you give me an example of that', 'how did/do you feel about that'.

Cancer diagnostic pathway

For peer review only

Initial questions about the referral pathway at the specific trust may not be needed if they have been covered in previous interviews.

Can you tell me about how the head and neck urgent cancer referral pathway is organised at the Trust?

- ***Establish in detail (who (including involvement of Nurses and AHPs), how, when):***
    - ***how referrals are processed***
    - ***how clinics are organised***
    - ***how onward referrals are made***

I'd like to know about changes that happened in the referral pathway during COVID-19 – how did you manage urgent referrals during that period?

- ***Did they use any means of triaging referrals? – if so find out about this in detail and about their experiences of this.***

Can you tell me about your role within the head and neck urgent referral cancer pathway?

The EVEREST study is aiming to improve the diagnostic pathway for urgent head and neck cancer referrals. It would be good to know from your point of view what is good about the current pathway and what could be improved.

- **What things do you think are good about the current diagnostic pathway for urgent referral?**

- **What things could be better?**

- **Are there particular sub-groups of patients that are more straightforward or harder to assess?**

You probably remember that we observed/recorded your consultation with (name). Would it be ok if we talked a bit about the consultation so that we can understand more about the processes that were happening?

For peer review only

Specific questions about e.g. why they pursued a particular line of questioning, or what triggered a decision to request a particular test

The EVEREST study is planning to introduce an intervention within the diagnostic pathway. Patients will be asked some questions about their symptoms either on a computer or on the phone. This would be triggered by the receipt of the referral at the hospital. Their answers would be used to calculate a risk score which would be made available to the hospital team, along with any additional information provided by the patient. We hope that this might enable the hospital team to make some decisions ahead of a hospital appointment, e.g. about tests or who needs to be seen most quickly and to provide relevant information in a standardised and accessible format.

What do you think of this idea?

- ***Feasible? (digital exclusion)***
- ***Useful? (an improvement? Would they trust a risk score?)***
- ***How would you see it working? (who might be involved – what roles might change? Who would need to be involved in planning for change?)***
- ***Add to or reduce workloads?***
- ***Is this the best way to improve the pathway or is there something that you think would work better?***

Anything Not Covered?

Is there anything that we haven't covered in the interview that you think we should know or think about?

Closing and Thanks

Conclude the discussion and thank the participant for their time and contribution.

For peer review only
